# SIRT1-SIRT7 Expression in Patients with Lymphoproliferative Disorders Undergoing Hematopoietic Stem Cell Mobilization

**DOI:** 10.3390/cancers14051213

**Published:** 2022-02-25

**Authors:** Mateusz Nowicki, Agnieszka Wierzbowska, Emilia Stec-Martyna, Dominika Kulczycka-Wojdala, Grzegorz Nowicki, Anna Szmigielska-Kapłon

**Affiliations:** 1 Department of Hematology, Copernicus Memorial Hospital in Lodz Comprehensive Cancer Center and Traumatology, 93-513 Lodz, Poland; agnieszka.wierzbowska@umed.lodz.pl (A.W.); anna.szmigielska-kaplon@umed.lodz.pl (A.S.-K.); 2 Department of Hematology, Medical University of Lodz, 93-513 Lodz, Poland; 3 Central Scientific Laboratory, Medical University of Lodz, 93-513 Lodz, Poland; emilia.stec-martyna@umed.lodz.pl (E.S.-M.); dominika.kulczycka-wojdala@umed.lodz.pl (D.K.-W.); 4 School of Continuing Studies, Georgetown University, Washington, DC 20001, USA; gln8@georgetown.edu

**Keywords:** sirtuins, multiple myeloma, lymphoma, hematopoietic stem cells, mobilization, autologous transplantation, apheresis

## Abstract

**Simple Summary:**

One of the treatment lines of patients with lymphoproliferative disorders is autologous transplantation, preceded by an effective mobilization of hematopoietic stem cells from the bone marrow niche into the peripheral blood. Sirtuins (SIRT1-SIRT7) are members of the NAD + -dependent class III histone deacetylase family, which affect the inflammatory response, hematopoiesis, angiogenesis, and the behavior of hematopoietic cells during mobilization and transplantation. In our work, we wanted to investigate how sirtuins expression changes on the day of the first apheresis as compared to the period before mobilization and how they can influence the efficacy of CD34+ cell collection. Our study revealed a significant increase in sirtuin expression on the day of the first apheresis as compared to the baseline. We demonstrated that the expression of SIRT5, SIRT6, and in particular SIRT7, on the day of first apheresis, may be associated with the efficacy of hematopoietic stem cell mobilization.

**Abstract:**

Sirtuins are involved in the fate of hematopoietic stem cells (HSCs), including their metabolism, stress response, differentiation, migration, and apoptosis. The aim of this study was to explore SIRT1-7 expression during HSC mobilization. The study included 50 patients with lymphoproliferative disorders (39 multiple myeloma, 11 lymphoma). Samples were taken before mobilization (day 0) and on the day of first apheresis (day A). The sirtuin expression was evaluated by the Droplet Digital PCR (ddPCR) method. A significant increase of the SIRT1, SIRT2, SIRT3, SIRT5, SIRT6, and SIRT7 levels measured at day A as compared to baseline was observed. The study revealed a positive correlation between SIRT5, SIRT6, and SIRT7 expression and the CD34+ peak value in peripheral blood and the number of CD34+ cells collected on day A. Patients from the SIRT7 “high expressors” group collected more CD34+ cells on day A than “low expressors”. Upregulated expressions of SIRT3 and SIRT7 on the day of first apheresis were observed in patients in complete remission status (CR) as compared to the non-CR group. Our results suggest that the investigated sirtuins may influence the HSC migration and hematopoietic landscape during mobilization. SIRT5, SIRT6, and SIRT7 may be associated with the efficacy of HSC mobilization.

## 1. Introduction

Lymphoproliferative disorders, such as multiple myeloma, Hodgkin, and non-Hodgkin lymphomas, are hematological malignancies in which abnormal proliferation of lymphoid lineage cells is observed [1,2]. Mobilization of hematopoietic stem cells (HSC) from the hematopoietic niche into the peripheral blood is a crucial step in the treatment of lymphoproliferative neoplasms with autologous hematopoietic stem cell transplantation (auto-HSCT) [3]. During HSC mobilization, patients receive chemotherapy and granulocyte colony-stimulating factor (G-CSF) or G-CSF in monotherapy [3,4]. This results in the release of CD34+ cells into the peripheral blood. In the next stage, the CD34+ cells are collected during the apheresis process. The number of HSCs harvested is important to increase the safety of the auto-HSCT procedure [5].

Factors and processes influencing mobilization efficiency are still not fully discovered and understood. It is known that the CD34+ cell migration is controlled by a complex milieu of components participating in HSC maintenance, survival, proliferation, and differentiation [4,6]. This includes intrinsic signaling pathways as well as the interaction of elements of the bone marrow niche mediated by adhesive molecules, growth factors, cytokines, proteases, miRNAs, and sirtuins [7,8,9].

Among these factors, sirtuins may play an important role in the mobilization of HSCs through deacetylating activity, which regulates many important processes related to the fate of HSCs, their metabolism, stress response, differentiation, aging, and apoptosis [10,11,12].

Sirtuins are members of the NAD+-dependent class III histone deacetylase family (SIRT1-SIRT7), involved in the post-translational modification of proteins, mainly through their deacetylation, polyADP-ribosylation, demalonylation, and desuccinylation [12,13]. The enzymatic activity of sirtuins depends on their mode of action and location in the cell. SIRT1 and SIRT2 are found both in the nucleus and the cytoplasm; SIRT6 and SIRT7 are present in the nucleus only; SIRT3, SIRT4, and SIRT5 are found in the mitochondria [13,14].

Sirtuins also play an important role in cancer development and resistance to chemotherapy [15,16]. The action of SIRT1 and SIRT2 is pivotal, depending on the type of tumor and the signaling pathway they affect [14,16]. In hematological malignancies, SIRT1 initiates and promotes the progression of neoplasms through deacetylation of the transcription factors p53, p73, or hypermethylation in cancer 1 protein (HIC1) [14,17,18]. Overexpression of SIRT1 promotes survival of cancer cells via inhibition of the synthesis of proteins and factors responsible for DNA repair and tumor suppressor factors as well as the inhibition of cell apoptosis. [11,14,19].

SIRT2 is considered to be a tumor suppressor, and under stress conditions can induce cell senescence or apoptosis [14,20]. Patients with overexpression of SIRT2 had upregulated expression of the genes responsible for the mitogen-activated protein kinase (MAPK) and vascular endothelial growth factor (VEGF) signaling pathways [21].

Sirtuins influence the effectiveness of chemotherapy by regulating reactive oxygen species (ROS) [22]. Most of the chemotherapy regimens induce an increase in intracellular levels of ROS, which results in damage to cancer cells and their apoptosis [22,23]. SIRT3 plays an important role in this process via acetylation of proteins in the mitochondrial environment and reduction of oxidative stress [11,24]. SIRT3 reduces oxidative stress in HSC cells, which leads to their prolonged survival and inhibits the aging process [25]. Knockout of this sirtuin downregulates the superoxide dismutase 2 (SOD2) activity [26]. SIRT1 affects the stemness of HSC by activating the forkhead box O (FOXO) pathway, inhibiting p53, but also by eliminating ROS [27,28].

Sirtuins significantly control the fate of HSCs, including their development, proliferation, migration from the marrow niche to the peripheral blood, and angiogenesis that occurs after the administration of mobilization chemotherapy [29,30,31]. SIRT7 deacetylates p53, which results in HSC proliferation and aging delay [32]. SIRT2 inhibits the p53 acetylation process [33]. SIRT1 may act pivotally as a negative regulator of the insulin-like growth factor 1 (IGF-1)/mTOR pathway and an inhibitor of the aging of HSC [34].

By controlling the transcriptional factors FOXO, sirtuins, especially SIRT1, influence the number of HSCs, their apoptosis, and the inhibition of the cell cycle [35,36]. Mice with SIRT1 knockout showed an increase in HSC proliferation and renewal capacity [30]. The decreased expression of SIRT3 in aged HSC is associated with myeloid biased differentiation, development of the stem cell pool, and decline in homing and engraftment potential [37]. SIRT6 deletion affects the dysfunction of the Wnt and NKB1 pathways, which is associated with increased expression of proinflammatory cytokines and further expansion of HSCs [38].

Myelopoiesis and granulopoiesis are activated in the course of the mobilization process and are regulated by sirtuins. SIRT1 inhibits the development of neutrophils through CCAAT/enhancer binding protein C/EBPs deacetylation, and the level of this sirtuin decreases with the progression of granulopoiesis [39]. SIRT3/SIRT5 expression gradually decreases with the differentiation of the common myeloid progenitor (CMP) to the granulocyte/monocyte progenitor (GMP) and from GMP to granulocytes [39]. A lack of SIRT6 results in a reduction of B cell progenitors in the bone marrow niche [40]. Increased numbers of granulocytes and T cells, as well as upregulated secretion of proinflammatory cytokines, were observed in mice with the SIRT7 knockout. It has been shown that high levels of NAD+ and G-CSF mediating the granulopoiesis-stimulating enzyme nicotinamide phosphoribosyltransferase (NAMPT) positively correlate with SIRT1 expression [10,41]. NAMPT promotes the myeloid differentiation of CD34+ cells. SIRT1 increases the expression of G-CSF and G-CSF receptor genes [10,41].

SIRT1 inhibitors delay differentiation and further increase HSC migration, including homing and CD34+ engraftment in response to stromal cell-derived factor 1 (SDF-1) [30]. Downregulation of SIRT1 expression promotes proliferation of cells expressing the vascular cell adhesion molecule 1 VCAM1, very late antigen-4 (VLA4), and lymphocyte function-associated antigen 1 (LFA-1) proteins, and does not change the number of cells with the CXCR4 receptor [30]. In AML cell cultures, the positive correlation between expression of SIRT1 and CXCR4 was found [42].

Sirtuins significantly affect the angiogenesis process. Both SIRT1 and SIRT2 often act in the opposite way [31,32,33,34,35,36,37,38,39,40,41,42,43,44,45,46,47,48]. SIRT3, by deacetylation of FOXO3, increases EC survival, especially in hypoxia, while overexpression of this sirtuin results in limited angiogenesis due to negative regulation of ROS [48]. Expression of SIRT6 positively influences the survival of EC cells and is proangiogenic. The function of SIRT7 in angiogenesis is pivotal. Upregulated levels of this sirtuin suppress the transforming growth factor-β (TGF-β) pathway and lead to the inhibition of the formation of new vessels and cancer metastasis [49]. On the other hand, expression of SIRT7 has been shown to promote angiogenesis through EC control and regulation of VEGF expression [50].

The bone marrow niche is a site of mutual processes between hematopoiesis and angiogenesis under physiological and stress conditions. It is worth exploring the role of sirtuins in these interactions.

The aim of our study was to investigate the role of sirtuins in HSC mobilization for autologous transplantation as well as to assess the impact of fluctuations in their expression on the efficiency of the CD34+ collection.

## 2. Materials and Methods

Fifty patients were enrolled in the study (24 F, 26 M). The median (Me) age was 60 years. The investigated group consisted of 39 multiple myeloma (MM), seven non-Hodgkin lymphoma (NHL) and four Hodgkin lymphoma (HL) patients. More comprehensive clinical data are presented in Table 1. Sirtuin expression was evaluated in peripheral blood (PB). The blood serum samples were collected at two time points: before hematopoietic stem cell mobilization (day 0) and on the day of the first apheresis (day A).

The blood was centrifuged at 1000x g for 10 min at 4 °C. Serum samples were stored frozen at −80 °C.

The mobilization regimens consisted of Cyclophosphamide, Cytarabine (Ara-C), and DCEP plus G-CSF or G-CSF in monotherapy for patients with MM, and ICE (R-ICE) or DHAP (R-DHAP) + G-CSF in lymphoma patients. Two patients with lymphoma received cytostatics in monotherapy: one cyclophosphamide and one AraC treatment.

Flow cytometry counting of CD34+ cells was assessed. Apheresis was performed using a Spectra Optia device. In patients mobilized with chemotherapy and the granulocyte growth stimulation factor (G-CSF), the median length of G-CSF administration until the first apheresis was nine days (range: 5–22).

The study was performed in accordance with the ethical standards of the Ethics Committee of the Medical University of Lodz and with the Helsinki Declaration.

### 2.1. RNA Extraction and cDNA Synthesis

RNA (including miRNA) was extracted from 200 μL of serum using the miRNeasy Serum/Plasma Mini Kit (.QIAGEN, Hilden, Germany, Cat. No. 217004) according to the manufacturer’s protocol. RNA samples (30 µL) were kept at −80 °C until cDNA synthesis by reverse transcription reaction. The cDNA was synthesized from the maximum volume (14 µL) of RNA using a Maxima First Strand cDNA Synthesis Kit for RT-qPCR (Cat. No. K1641, Thermo Scientific, Waltham, MA, USA). mRNA levels (copies/µL) of the SIRT2 and SIRT5 are presented in Figure S1.

### 2.2. Absolute Gene Expression with Digital Quantitative PCR

Gene expression analysis from the serum was obtained with digital quantitative PCR using specific ddPCRTM Gene Expression Assays (Bio-Rad Inc., Hercules, CA, USA) and a QX200 droplet digital PCR system (Bio-Rad) according to the manufacturer’s instructions. The following probes were used: dHsaCPE5033410 for SIRT1, dHsaCPE5057130 for SIRT2, dHsaCPE5027422 for SIRT3, dHsaCPE5052282 for SIRT4, dHsaCPE5053664 for SIRT5, dHsaCPE5052126 for SIRT6, and dHsaCPE5027454 for SIRT7 (Bio-Rad). The ddPCR mixture was composed of 11 µL of 2 × ddPCR Supermix for Probes (no dUTP) (Bio-Rad, Hercules, CA, USA, Cat. No. 1863024), 1.1 µL ddPCR™ Gene Expression Assay (Bio-Rad), which consisted of primers and an FAM-labeled fluorescent probe, 8.9 µL DNase/RNase free MilliQ water, and 1 µL of template DNA in a final reaction volume of 22 μL. Droplets were then generated in the QX200 droplet generator (Bio-Rad) by loading 20 μL of the reaction mixture and 70 μL of droplet generation oil for probes (Bio-Rad) onto matched wells of a DG8 cartridge (Bio-Rad). Then, 40 μL of the droplet/oil mixture was transferred to a semi-skirted 96-well plate (Bio-Rad). The plate was then heat-sealed with a pierceable aluminum foil using a PX1 PCR plate sealer (Bio-Rad) set to run at 180 °C for 5 s. The PCR was performed in a T100 Thermal Cycler (Bio-Rad). The PCR thermal cycling conditions are presented in Table S2.

The fluorescence signals were measured by the QX200 Droplet Reader (Bio-Rad). The positive droplets containing amplified products were discriminated from negative droplets by applying a threshold above the negative droplets. Reactions with more than 10,000 accepted droplets per well were used for analysis using QuantaSoft™ Analysis Pro software version 1.0.596 (Bio-Rad, Hercules, CA, USA). Subsequently, obtained results were converted into copies/200 µL of the input material regarding the input serum volume and dilutions on the level of RT and PCR reaction. Representative 1D plot of ddPCR reactions for SIRT2 and SIRT5 are presented in Figure S2.

### 2.3. Statistical Analysis

The Wilcoxon matched-pairs test was used to compare groups of dependent continuous variables: sirtuin expression at two different time points. Correlations between variables were assessed by the Spearman rank correlation coefficient (R). The Mann–Whitney U-test was used to compare independent variables: number of collected CD34+ cells during the first apheresis and sirtuin expression (copies/200 µL serum). A least-squares (OLS) regression was performed to identify treatments influencing the amount of CD34+ cells in peripheral blood in patients on day A. Additionally, a simple linear regression was performed to further examine the relationship between expression of SIRT7 on day A and circulating CD34+ peak in morning blood. Comparisons and correlations were considered significant if *p* < 0.05. Statistical analysis was done using the Statistica 13.3 software and Python 3.7.12 in the Jupyter Notebooks environment.

## 3. Results

### 3.1. Mobilization Data

The median number of CD34+ cells collected after the first apheresis was 3.35 × 10^6^/kg. The median number of total collected CD34+ cells during mobilization was 5.23 × 10^6^/kg. The median number of CD34+ cells estimated in peripheral blood on the day of the first apheresis was 54.4/µL. The median number of apheresis attempts needed to collect at least 2 × 10^6^/kg CD34+ was 2. Detailed mobilization data of the patients enrolled in the study are presented in Table 2.

### 3.2. Kinetics of Sirtuins

The levels of SIRT5, SIRT6, and SIRT7 were mostly undetected on day 0 (Me: 0 copies/200 µL serum). The SIRT1, SIRT2, SIRT3, SIRT5, SIRT6, and SIRT7 levels measured at day A were significantly increased as compared to day 0 (Table 3). Moreover, the expression of SIRT4 was mostly undetected at both time points (Me: 0 copies/200 µL serum). SIRT4 was detected only in one patient on day 0 and in seven patients on day A. The level of sirtuins expression in patients during mobilization is presented in Table S1

Delta values were calculated to measure the relative changes in sirtuin levels between day 0 and day A. For every sirtuin, the average of Day 0 data was subtracted from the mean of the data collected on Day A. The following results were obtained: SIRT1 = 766.77, SIRT2 = 841.84, SIRT3 = 536.93, SIRT5 = 271.4, SIRT6 = 357.60, and SIRT7 = 482.06. Calculating the Delta for SIRT4 did not yield significant results. It should be noted that the delta of SIRT2 (841.84) was the highest among the researched SIRTs.

### 3.3. Sirtuin Level and Mobilization Efficacy

To assess the effectiveness of mobilization, sirtuin level was assessed against (1) the number of CD34+ cells in peripheral blood at the first apheresis, (2) the number of collected CD34+ cells on the day of the first apheresis, (3) the total number of CD34+ cells collected during mobilization and (4) the number of apheresis attempts.

#### 3.3.1. Sirtuin Expression and the CD34+ Peak Value in Peripheral Blood at First Apheresis

**Figure 1:**
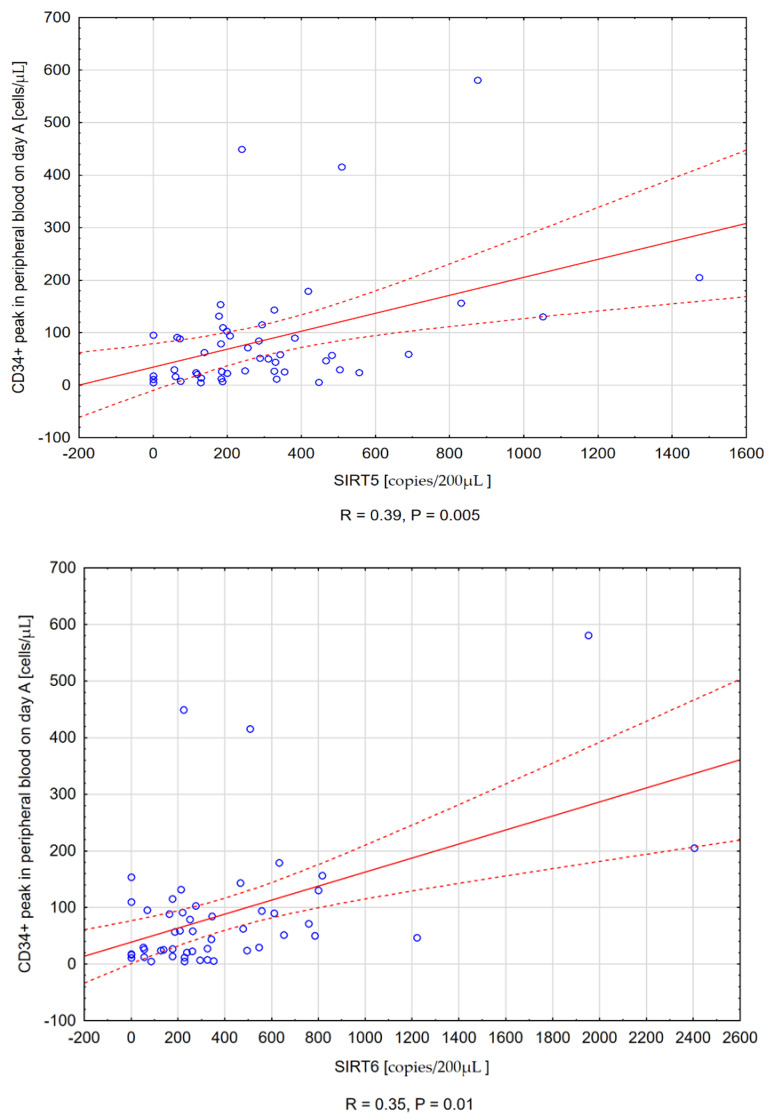

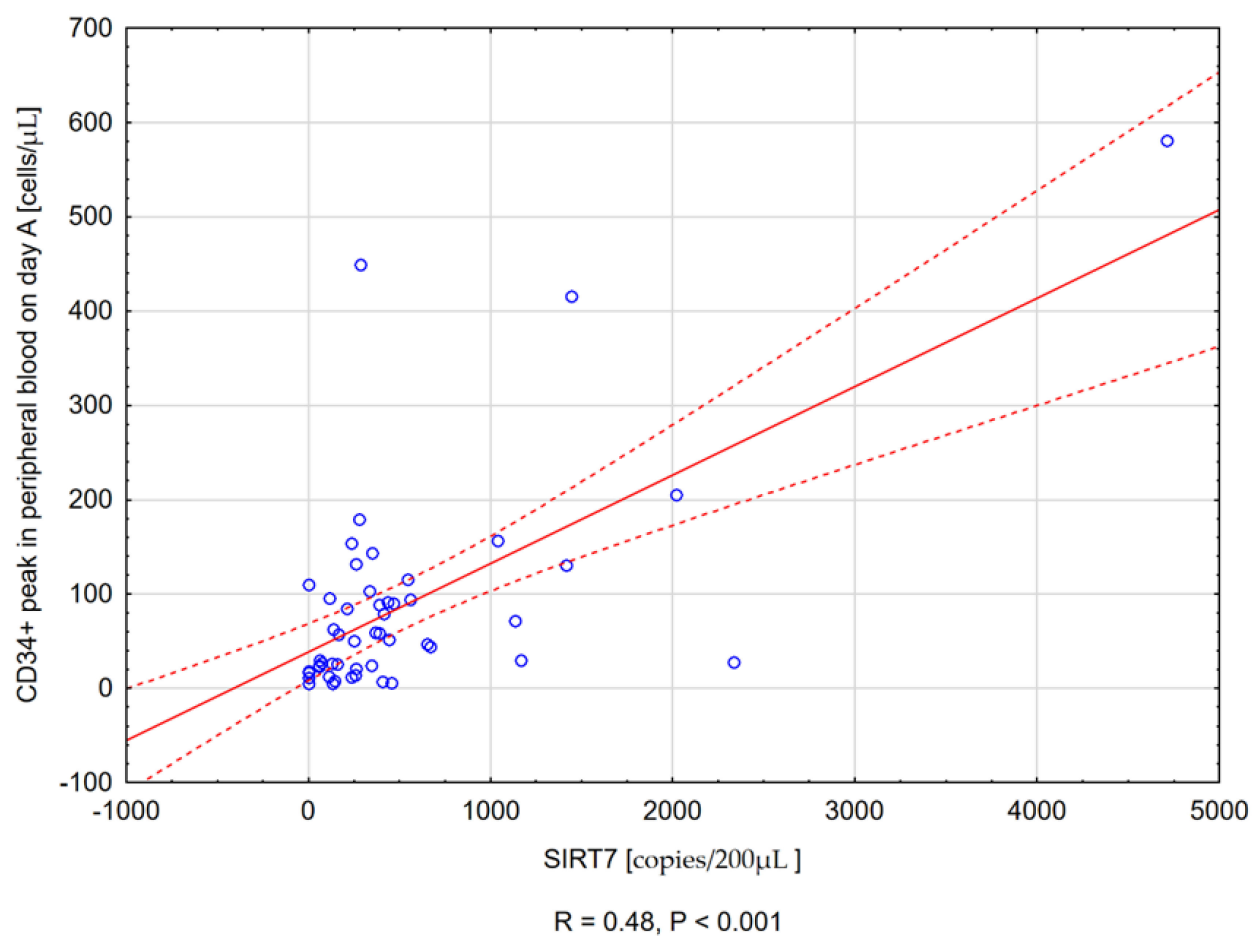
Scatter plots illustrating the positive correlation between sirtuin expression on day A and CD34+ peak in peripheral blood. A - SIRT5, B - SIRT6, C - SIRT7.

Positive correlations were observed between SIRT5, SIRT6, and SIRT7 levels on day A and CD34+ number in the peripheral blood at the same time point (R = 0.39, *p* = 0.005), (R = 0.35, *p* = 0.01), and (R = 0.48, *p* < 0.001), respectively (Figure 1).

An ordinary least-squares (OLS) regression was performed to identify treatments influencing the number of CD34+ cells in the morning peripheral blood in patients. The clinical factors taken into consideration were: age; SIRT5, SIRT6, and SIRT7 levels; and white blood cell (WBC) count on day A. Among the included factors, only SIRT7 level exhibited a connection to the level of CD34+ peak in peripheral blood (Table 4). It is worth mentioning that the regression result for WBC, although not as significant as that of SIRT7, might warrant further analysis. A simple linear regression was performed to further examine the relationship between SIRT7 expression and the number of CD34+ cells in peripheral blood on day A. This further confirmed the statistical significance of this connection through a lower standard error and *p*-value. The results of the simple linear regression are presented in Table 5.

#### 3.3.2. Sirtuin Concentration and the Number of Collected CD34+ Cells on the Day of First Apheresis

Positive correlation was noticed between SIRT5, SIRT6, and SIRT7 expression on day A and the number of CD34+ cells collected at the first apheresis (R = 0.34, *p* = 0.02), (R = 0.31, *p* = 0.03), and (R = 0.47, *p* < 0.001). No correlation was observed between sirtuin concentration on day 0 and the number of collected CD34+ cells at the first apheresis.

To evaluate the influence of sirtuin expression on the number of CD34+ cells on day A, patients were divided into “high” and “low” expression groups according to median sirtuin levels on the day of first apheresis (above and below median). The group of SIRT7 “high expressors” collected more CD34+x10^6^/kg cells on day A than “low expressors” (5.01 vs. 1.68 CD34+ ×10^6^/kg, *p* = 0.003).

#### 3.3.3. Sirtuin Expression and the Total Number of CD34+ Cells Collected during Mobilization

No correlation was found between sirtuin levels on day A and the total number of collected CD34+ cells. Additionally, no statistically significant differences were found in the total number of CD34+ collected cells between “high” and “low” expressors.

#### 3.3.4. Sirtuin Level and the Number of Apheresis Attempts

**Figure 2:**
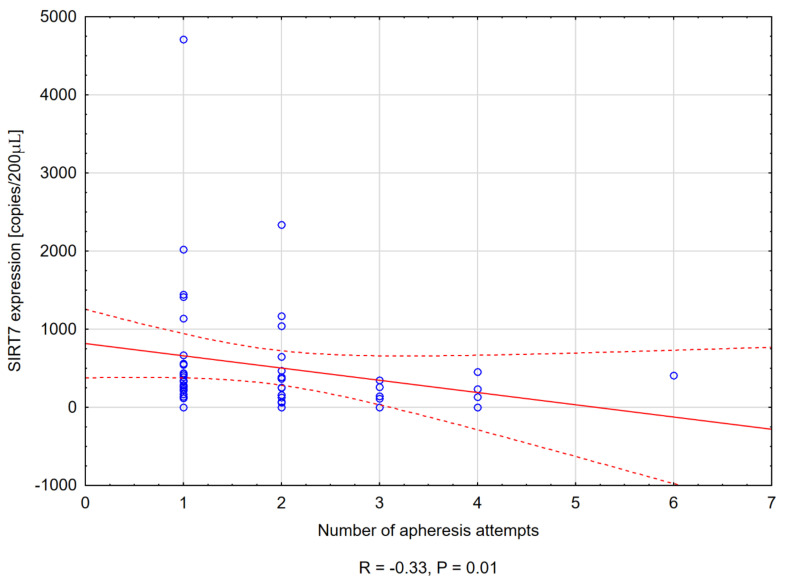
Scatter plot presenting the negative correlation between SIRT7 expression on day A and the number of apheresis attempts needed to collect at least 2 × 10^6^/kg of CD34+ cells.

Only for SIRT7, a negative correlation was noticed between its expression on day A and the number of apheresis attempts (R = −0.33, *p* = 0.01) (Figure 2).

### 3.4. Relationship between WBC and Sirtuins

The sirtuin expression was correlated with WBC on the day of the first apheresis. A positive correlation was observed between SIRT1, SIRT2, SIRT3, SIRT5, SIRT6, and SIRT7 and WBC (R = 0.47, *p* < 0.001), (R = 0.60, *p* < 0.001), (R = 0.49, *p* < 0.001), (R = 0.41 *p* = 0.002), (R = 0.48, *p* < 0.001), and (R = 0.44, *p* = 0.001), respectively. 

### 3.5. Sirtuin Levels and Remission Status

The association between sirtuin concentration on day A and the depth of myeloma/lymphoma response (CR versus not CR before mobilization regimen) was evaluated. Patients mobilized with chemotherapy and G-CSF (n = 45) were divided into CR (n = 10) and non-CR (n = 35) groups. The CR group had higher SIRT3 and SIRT7 levels on the day of the first apheresis than the non-CR group (Me = 369.04 vs. 136.47 copies/200 µL, *p* = 0.03), (Me = 435.65 vs. 258.22 copies/200 µL, *p* = 0.04), respectively.

## 4. Discussion

Sirtuins have a significant impact on the development of hematological malignancies, as well as on the balance of the bone marrow niche, under both physiological and stressful conditions. Considering their role in the cell cycle, changes in sirtuin expression may influence the migration of HSC, both homing after transplantation and the release of these cells from the hematopoietic niche during mobilization [10,11,12]. To our knowledge, there is no existing research on the expression of these enzymes in patients with lymphoproliferative disorders undergoing CD34+ cell mobilization. Furthermore, the influence of sirtuins on the effectiveness of this process has not been investigated. 

In our study, we observed a significant increase in the expression of SIRT1, SIRT2, SIRT3, SIRT5, SIRT6, and SIRT7 on the day of first apheresis compared to the day before mobilization. It seems that the elevated expression of these sirtuins is associated with the use of chemotherapy during mobilization, which disturbs the environment of the bone marrow niche and causes stress-induced hematopoiesis. Chemotherapy agents are stress factors that damage progenitor cells and daughter cells and induce expansion of the HSCs. Chemotherapy stress activates oxidative phosphorylation in the mitochondria [52]. This may explain the significant increase in expression of the sirtuins, mainly present in the mitochondria: SIRT3 and SIRT5. Of particular importance is the role of SIRT3, as it regulates the acetylation landscape of mitochondrial proteins during oxidative stress that occurs after chemotherapy [53]. Moreover, the increased expression of SIRT3 is mainly observed in young HSC cells, which are present in peripheral blood during mobilization and apheresis [25].

In our work, despite mobilization with chemotherapy and the assumed decrease in NAD+, we observed a significant increase in sirtuin expression. The deacetylating activity of sirtuins is dependent on the level of the NAD+ cofactor [12,13]. Interestingly, the level of cellular NAD+ decreases in aging cells, cancer cells, or when exposed to stress factors such as chemotherapeutic agents [54]. We suppose that our results may be related to the G-CSF administration during the mobilization process. In some of the patients from our study, only G-CSF in monotherapy was used in mobilization, and an increase in sirtuin expression was also observed in this group. It has been shown that intracellular levels of NAD+ and NAMPT in myeloid cells, as well as their plasma levels, increased in both healthy volunteers and neutropenic patients [10]. NAMPT, which generates NAD+, is required for G-CSF-induced myeloid differentiation of CD34+ cells. It was previously found that increased SIRT1 may be related to increased NAMPT expression [10]. 

Upregulated expression of SIRT1 on day A may be related to deacetylation of p53 and FOXO1 after chemotherapy, which promotes angiogenesis promoting [31]. Moreover, SIRT1 inhibits the anti-angiogenic NOTCH pathway, which is responsible for maintaining the HSC population by promoting quiescence and inhibiting their maturation [44]. High expression of SIRT2 after chemotherapy also influences angiogenesis by regulating EC survival and promoting cytoskeleton remodeling in the EC [50]. SIRT2 plays an important role in HSC maintenance. Its upregulated level is observed in young HSCs. [55] Additionally, a high level of SIRT3 increases EC survival, especially under hypoxic conditions through deacetylation of FOXO3 [48].

Upregulated SIRT2 was observed in patients with overexpressed genes, responsible for MAPK and VEGF signaling pathways [21]. G-CSF activates the MAPK signaling axis, which is responsible for neutrophil progenitor proliferation [56]. It may explain the positive correlation between SIRT2 level and WBC on the day of first apheresis.

The observed increased expression of SIRT3 and SIRT5 on the day of the first apheresis may be associated with the initial intensive reconstruction of the bone marrow niche after chemotherapy. As regeneration progresses, a gradual decline in the expression of these sirtuins is observed during the differentiation from CMP to GMP and from GMP into granulocytes, reflecting the decrease in mitochondrial DNA during subsequent stages of hematopoiesis [39]. It also may explain the positive correlation between SIRT3, SIRT5, and WBC count on the day of first apheresis. 

In our study, positive correlations were found between SIRT5, SIRT6, and SIRT7 and the CD34+ peak value in peripheral blood, as well as the number of CD34+ cells collected at first apheresis. 

SIRT5 has a positive effect on the aforementioned early stage of hematopoiesis. SIRT6 protects EC cells from DNA damage and telomere dysfunction [29,57]. It has been shown that the lack of SIRT7 negatively affects the regenerative capacity of HSCs [58]. In transplant recipients, HSCs significantly lose the ability to regenerate the hematopoietic niche as compared to cells in which this sirtuin is present [57]. Moreover, SIRT7 promotes angiogenesis by modulating EC function and regulating VEGF expression [29,50]. This may also explain why patients with higher SIRT7 expression obtained significantly more CD34+ cells on the day of the first apheresis compared to patients with lower expression. The impact of this sirtuin on the HSC cycle also reflects that its upregulated expression in our patients results in fewer apheresis attempts needed to collect at least 2 × 10^6^/kg CD34+ cells.

Interestingly, we found no relationship between the levels of SIRT5, SIRT6, and SIRT7 and the total amount of CD34+ cells collected during mobilization. These sirtuins were also undetectable in the majority of patients in the pre-mobilization period. Previous studies have shown that the lack of SIRT6 promotes the proliferation of HSCs by activating the Wnt pathway [38]. The deletion of SIRT6 causes activation of the NF-κB signaling pathway by regulating the expression of the L1 Cell Adhesion Molecule (LCAM1), NFKB1, and genes encoding proinflammatory cytokines, which in turn influence the expansion of HSCs [38]. In mice with the SIRT7 knockout, acetylation and increased p53 activity was observed [59]. This promotes apoptosis and reduced resistance to stress and compromises the regenerative capacity of HSCs [58,59]. These data suggest that the action of sirtuins is pivotal and that their role in the context of mobilization is ambiguous.

In our study, we did not find a statistically significant difference in SIRT4 expression between the day before mobilization and the day of the first apheresis. Moreover, the expression of SIRT4 was mainly at an undetectable level. Previous studies have found that the expression of this sirtuin is downregulated in many cancers, including thyroid, colon, bladder, breast, stomach, and ovarian cancers, and it has been associated with a worse prognosis [60]. Moreover, suppression of SIRT4 may increase the level of pro-inflammatory cytokines [61].

This may explain the lack of expression of this sirtuin on the day of the first apheresis. Chemotherapy combined with G-CSF stimulation causes inflammation associated with intense angiogenesis and disruption of signaling pathways associated with HSC motility [61,62].

Interestingly, in murine models with Burkitt lymphoma, the SIRT4 knockout resulted in accelerated lymphomagenesis and increased mortality [63]. It should be mentioned that failure to detect SIRT4 may also be related to its characteristics. Unlike other sirtuins, SIRT4 has NAD+-dependent ADP-ribosyltransferase activity; its enzymatic action is much weaker than in other sirtuins, and hence it is very hard to detect [60].

Increased expression of SIRT3 has been observed in patients with CR. Complete remission is the result of the increased effectiveness of the applied chemotherapy, which results in enhanced angiogenesis and the formation of reactive oxygen species [22,25,64]. It should be emphasized that the use of G-CSF both in monotherapy and together with chemotherapy also promotes the formation of reactive oxygen species [63]. ROS promotes tumor cell damage and apoptosis, and the effectiveness of these processes may be influenced by SIRT3 expression [23,25,26]. It is worth noting that the CR group included patients with both multiple myeloma and lymphoma who received other chemotherapy regimens (cyclophosphamide, cytarabine, R-DHAP, R- ICE). We suppose that SIRT3 overexpression may be an individual feature, and an analysis should be performed on a larger group of patients, and separately for patients with different chemotherapy regimens.

Upregulated expression of SIRT7 in patients in CR may be associated with increased angiogenesis and ongoing reconstitution of the bone marrow niche after chemotherapy [65]. It is worth noting that SIRT7 stabilizes p53, which is responsible for cell-cycle arrest in response to stress, facilitates DNA repair, and triggers activation of apoptosis to eliminate damaged cells [66,67]. These processes are especially important in achieving complete remission after the applied treatment.

## 5. Conclusions

In conclusion, we observed that higher levels of certain sirtuins are associated with the efficacy of CD34+ cell mobilization for autologous transplantation. SIRT5, SIRT6, and SIRT7 play a special role in this process. Fluctuations in the expression of sirtuins were observed, depending on the remission status of the patients. We are aware of the limitations of our work and believe that further studies should be conducted on a larger group of patients. Furthermore, we recommend that the fluctuations in sirtuin expression in individual lymphoproliferative diseases need to be investigated separately.

## Figures and Tables

**Table 1:** Characteristics of the patients enrolled in the study.

Characteristics	Numbers
Age (years)	Median 60 (range 44–69)
Sex (female/male)	24/26
Multiple myeloma	39 (7 CR, 24 VGPR, 8 PR)
Hodgkin lymphoma	4 (1 CR, 3 PR)
non-Hodgkin lymphoma: Diffuse large B-cell lymphoma Mantle cell lymphoma Anaplastic large-cell lymphoma Hepatosplenic T-cell lymphoma	7 3 (1 CR, 2 PR) 2 (CR) 1 (PR) 1 (PR)
Mobilization chemotherapy: Multiple myeloma Hodgkin and non-Hodgkin lymphoma	Cyclophosphamide (2000 mg/m^2^) (n = 26) DCEP (Dexamethasone 40 mg, Cyclophosphamide 400 mg/m^2^, Cisplatin 10 mg/m^2^, Etoposide 40 mg/m^2^) (n = 6) Cytarabine (1600 mg/m^2^) (n = 2) G-CSF (10 µg/kg) in monotherapy (n = 5) ICE (Ifosfamide 3000 mg/m^2^, Carboplatin AUC = 5, max 800 mg/m^2^, Etoposide 100 mg/m^2^) (n = 5)R-ICE (with rituximab 375 mg/m^2^) (n = 1) DHAP (Dexamethasone 40 mg, Cytarabine 2000 mg/m^2^, Cisplatin 100 mg/m^2^) (n = 1) R-DHAP (with rituximab 375 mg/m^2^) (n = 2) Cyclophosphamide (2000 mg/m^2^) (n = 1) Cytarabine (1600 mg/m^2^ (n = 1)

Best response achieved prior to mobilization procedure: CR—complete remission, VGPR —very good partial remission (only multiple myeloma), PR—partial remission.

**Table 2:** Clinical data on hematopoietic stem cell (HSC) mobilization in patients enrolled in the study.

Characteristics	Numbers
CD34+ cells collected during mobilization (total number) (×10^6^/kg)	Median 5.23 (range 2.2–35.6)
CD34+ collected on Day A (×10^6^/kg)	Median 3.35 (range 0.3–35.6)
Number of apheresis needed to collect at least 2 × 10^6^/kg CD34+	Median 2 (range 1–6)
CD34+ peak in peripheral blood before first apheresis (cells/µL)	Median 54.4 (range 4.8–581)
WBC count on Day A (×10^3^/µL)	Median 16.91 (range 2.68–47.42)
Mobilization efficacy	
Good mobilizers	45
* Poor mobilizers	5

* After adequate mobilization (G-CSF 10 µg/kg if used alone or ≥5 µg/kg after chemotherapy), the circulating CD34+ cell peak was <20/µL up to 6 days after mobilization with G-CSF or up to 20 days after chemotherapy and G-CSF, or they yielded <2 × 10^6^ CD34+ cells per kg in ≤3 aphereses [51].

**Table 3:** Alterations in sirtuin expression (copies/200 µL serum, median values) before mobilization regimen (day 0) and on the day of the first apheresis (day A).

Sirtuin	Day 0 (Me, Range)	Day A (Me, Range)	*p* Value
SIRT1	68.55 range: 0–394.57	368.22 0–16748.56	*p* < 0.001
SIRT2	80.12 0–425.65	730.74 65.6–4787.37	*p* < 0.001
SIRT3	28.67 0–256.72	161.28 0–14167.35	*p* < 0.001
SIRT4	0 0–71.86	0 0–134.96	*p* = 0.05
SIRT5	0 0–255.69	243.6 0–1473.39	*p* < 0.001
SIRT6	0 0–240.22	256.16 0–2404.06	*p* < 0.001
SIRT7	0 0–206.93	282.96 0–4711.99	*p* < 0.001

**Table 4:** Results of OLS regression performed to examine how CD34+ peak value on the day of first apheresis is influenced by age, SIRT5, SIRT6, and SIRT7 expression, and white blood cell (WBC) count on day A.

Factor	Coeff	Std Err	t	*p* > ǀtǀ	[0.025	0.975]
Intercept	1.8104	79.591	0.023	0.982	−158.594	162.215
Age	1.2544	1.380	0.909	0.368	−1.527	4.036
SIRT5 (Day A)	0.0452	0.074	0.608	0.546	−0.105	0.195
SIRT6 (Day A)	0.0355	0.058	0.615	0.542	−0.081	0.152
SIRT 7 (Day A)	0.0911	0.025	3.611	0.001	0.040	0.142
WBC count (Day A)	−3.0984	1.395	−2.221	0.032	−5.909	−0.287

**Table 5:** Results of a simple linear regression testing the relationship between expression of SIRT7 on day A and the CD34+ peak value in peripheral blood on the day of first apheresis.

Factor	Coeff	Std Err	t	*p* > ǀtǀ	[0.025	0.975]
Intercept	38.3839	14.983	2.562	0.014	8.258	68.510
SIRT7 (Day A)	0.0939	0.016	5.861	0.000	−0.105	0.195

## Data Availability

The data presented in this study are available in Supplementary materials.

## Supplementary Materials

The following supporting information can be downloaded at: https://www.mdpi.com/article/10.3390/cancers14051213/s1, Figure S1: mRNA level [copies/µL] of the (a) SIRT2 and (b) SIRT5.; Figure S2: Representative 1-D plot of ddPCR reactions for (a) SIRT2 and (b) SIRT5 target genes.; Table S1: Clinical characteristics of the patients enrolled in the study along with the SIRT1-SIRT7 expression determined in patients on day 0 and day A.; Table S2: The PCR thermal cycling conditions (T100 Thermal Cycler, BioRad).
