# Supplementary material for: SIRT1-SIRT7 Expression in Patients with Lymphoproliferative Disorders Undergoing Hematopoietic Stem Cell Mobilization
Source: Cancers (Basel). 2022 Feb 25;14(5):1213. doi: 10.3390/cancers14051213 (PMC8909005; doi:10.3390/cancers14051213)
Supplement: Supplementary file 1 [file cancers-14-01213-s001.zip › Figure S2.pdf]

(a)

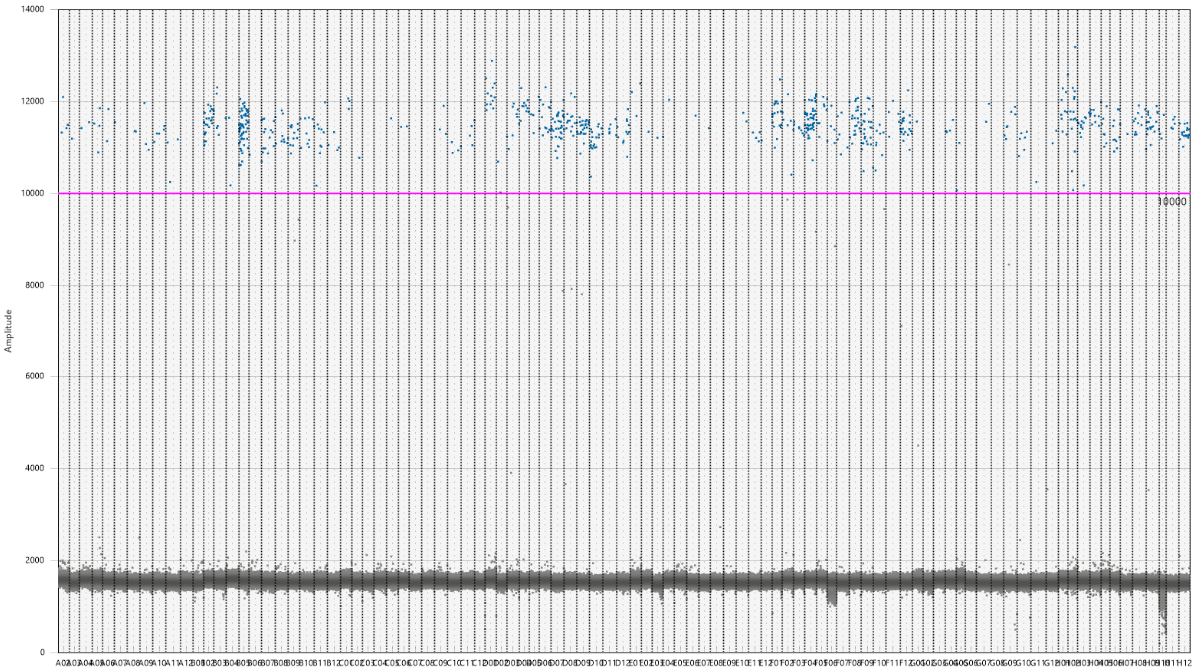

(b)

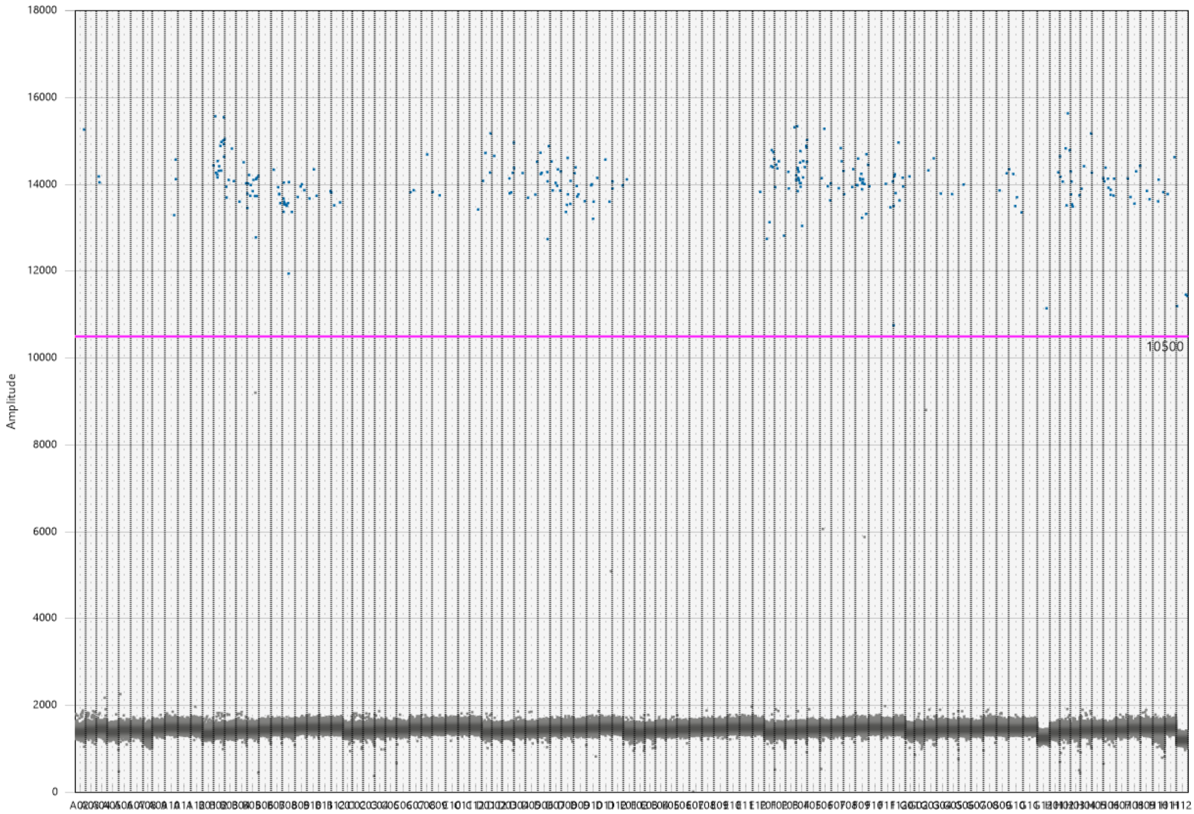

Figure S2: Representative 1-D plot of ddPCR reactions for (a) SIRT2 and (b) SIRT5 target genes. The ordinate scales indicate fluorescent amplitude. Blue dots indicate the positive droplets containing at least one copy of the target gene and gray dots indicate the negative droplets without any target gene.
