# Supplementary material for: SIRT1-SIRT7 Expression in Patients with Lymphoproliferative Disorders Undergoing Hematopoietic Stem Cell Mobilization
Source: Cancers (Basel). 2022 Feb 25;14(5):1213. doi: 10.3390/cancers14051213 (PMC8909005; doi:10.3390/cancers14051213)
Supplement: Supplementary file 1 [file cancers-14-01213-s001.zip › Table S1.pdf]

Table S1. Clinical characteristics of the patients enrolled in the study along with the SIRT1-SIRT7 expression determined in patients on day 0 and day A.

Patient number	Sex	Age	Diagnosis	Response 1 –CR, 0 – not CR	Number of apheresis	Number of CD34+ cells on day A [$x10^6$/kg]	CD34+/µl peak in peripheral blood on day A	Total number of collected CD34+ cells [$x10^6$/kg]	WBC in peripheral blood at day A[$x10^3$/µl]	Sirtuins expression on Day 0							Sirtuins expression on Day A						
										SIRT1	SIRT2	SIRT3	SIRT4	SIRT5	SIRT6	SIRT7	SIRT1	SIRT2	SIRT3	SIRT4	SIRT5	SIRT6	SIRT7
1	M	62	MM	CR	1	7.1	115.4	7.1	20.39	199.64	0	0	0	0	0	0	822.74	722.61	433.31	0	293.87	175.63	544.98
2	F	57	MM	CR	3	0.9	12.3	3.05	33.36	0	134.43	0	0	58.36	0	68.63	275.33	1309.42	136.83	74.65	183.7	56.24	112.46
3	M	65	MM	CR	1	4.97	103.2	4.97	23.3	0	0	71.77	0	60.54	0	60.93	420.87	721.23	190.5	0	198.21	275.63	334.78
4	M	63	MM	CR	4	0.32	5.5	2.5	27.31	145.81	0	0	0	120.29	0	68.18	302.26	859.37	478.6	0	446.43	351.47	455.98
5	M	48	MM	CR	1	7.27	94	7.27	9.4	137.66	75.22	74.14	0	0	0	139.2	561.16	660.21	453.75	0	206.67	557.09	559.72
6	F	51	MM	CR	1	4.25	56.8	4.25	17.64	76.86	0	80.44	71.86	0	0	81.74	1186.49	1369.18	503.86	0	482.01	186.97	166.57
7	M	60	MM	CR	1	10.8	88.8	10.8	14.85	0	77.73	0	0	0	0	75.25	0	155.33	155.95	66.51	71.75	163.75	389.19
8	F	61	MM	not CR (VGPR)	2	3.2	58.4	5.58	17.04	0	190.4	0	0	109.3	73.4	0	827.94	1615.35	678.65	58.13	342.31	261.86	389.85
9	F	65	MM	not CR (VGPR)	2	1.5	25.3	2.91	13.44	0	0	66.47	0	68.98	0	0	470.13	902.26	67.18	0	354.16	138.5	157.76
10	M	67	MM	not CR (VGPR)	6	0.82	7.4	2.99	30.85	0	0	0	0	0	128.22	0	621.94	1595.95	203.97	0	187.39	294.4	406.59
11	F	54	MM	not CR (VGPR)	2	1.7	24.2	24.3	3.16	0	242.39	57.34	0	0	0	58.32	367.98	357.15	176.89	0	115.87	125.95	60.39
12	F	39	MM	not CR (VGPR)	2	2.7	26.5	5.88	7.71	64.89	260.62	62.15	0	0	52.96	0	120.29	284.83	187.3	0	184.69	55.75	130.89

Table S1. Clinical characteristics of the patients enrolled in the study along with the SIRT1-SIRT7 expression determined in patients on day 0 and day A.

13	F	51	MM	not CR (VGPR)	1	4.52	132.2	4.52	26.28	126.29	69.36	72.72	0	0	0	0	312.71	330.27	285.18	0	177.68	212.48	260.82
14	F	57	MM	not CR (VGPR)	1	4.77	44.3	4.77	14.48	128.66	63.97	62.27	0	0	0	0	412.26	897.13	136.47	0	329.9	341.77	669.45
15	M	65	MM	not CR (VGPR)	2	4.59	59.3	11.58	9.83	237.68	128.69	0	0	0	55.48	79.37	122.6	576.36	66.85	0	689.03	208.18	367.45
16	F	62	MM	not CR (VGPR)	1	8.7	109.7	8.7	26.63	66.65	0	0	0	0	0	206.93	117.52	250.3	0	0	188.08	0	0
17	F	69	MM	not CR (VGPR)	1	6.1	91.2	6.1	20.78	0	0	0	0	59.14	0	0	293.33	1157.79	471.18	0	64.09	218.69	436.03
18	F	42	MM	not CR (VGPR)	3	1.15	11.34	4.5	6.75	0	109.85	195.53	0	0	0	0	73.3	67.19	0	0	0	0	0
19	M	63	MM	not CR (VGPR)	1	13.5	179.3	13.5	17.19	122.62	153.64	68.78	0	0	69.7	0	61.85	1050.31	143.95	67.12	418.61	631.5	280.81
20	M	50	MM	not CR (VGPR)	2	2.49	89.9	5.29	18.82	259.68	203.5	0	0	0	68.08	0	298.79	1147.41	0	0	381.85	611.3	468.82
21	F	51	MM	not CR (VGPR)	4	0.3	5.1	2.66	21.99	0	181.32	0	0	68.69	0	0	204.84	653.08	73.32	0	0	226.99	0
22	M	60	MM	not CR (VGPR)	1	3.5	51.9	3.5	16.56	71.79	217.46	0	0	0	0	0	369.82	871.5	0	0	288.63	652	441.44
23	M	60	MM	not CR (VGPR)	1	20.8	415.4	20.8	14.45	76.92	0	163.46	0	58.96	0	131.99	958.95	876.77	311.81	0	509.08	508.05	1445.53
24	M	55	MM	not CR (VGPR)	1	5.69	143.5	5.69	10.93	136.39	82.5	0	0	0	58.72	0	220.38	463.83	0	0	327.01	465.74	349.37
25	M	50	MM	not CR (VGPR)	1	21	449.5	21	9.79	394.57	72.5	256.72	0	0	56.39	0	263.39	385.28	132.43	0	239.33	224.94	285.1
26	F	63	MM	not CR (VGPR)	4	0.6	4.8	2.2	14.35	0	0	0	0	0	0	59.59	176.1	825.75	0	0	128.66	85.44	134.6
27	F	62	MM	not CR (VGPR)	4	0.78	11.9	3.1	15.65	229.99	76.41	0	0	0	73.49	0	527.82	436.63	240.91	0	333.19	226.95	236.28

Table S1. Clinical characteristics of the patients enrolled in the study along with the SIRT1-SIRT7 expression determined in patients on day 0 and day A.

28	F	60	MM	not CR (VGPR)	3	0.46	7.59	3.6	22.33	0	77.2	80.65	0	81.77	0	62.16	403.41	772.42	166.61	0	73.96	325.64	145.1
29	M	66	MM	not CR (VGPR)	1	35.6	581	35.6	40.86	69.87	319.29	68.18	0	0	68.24	0	16748.56	4787.37	14167.35	134.96	875.46	1951.47	4711.99
30	F	62	MM	not CR (VGPR)	1	7.5	130.4	7.5	47.42	210.98	299.12	149.56	0	71.14	240.22	142.3	1237.4	2378.59	644.96	0	1051.72	799.54	1415.21
31	F	60	MM	not CR (VGPR)	1	5.3	71.5	5.3	19.44	79.18	299.61	0	0	65.89	0	145.14	1789.7	738.86	748.4	0	255.37	757.53	1136.45
32	F	55	MM	not CR (PR)	2	0.9	14.3	3.42	4.13	0	54.05	122.8	0	0	0	0	64.75	322.95	0	0	129.75	177.07	258.22
33	M	40	MM	not CR (PR)	1	4.7	62.6	4.7	12.6	0	70.03	0	0	133.81	64.42	0	553.83	573.97	329.21	0	137.72	477.48	138.23
34	M	56	MM	not CR (PR)	2	2.62	27.6	4.46	28.77	178.93	193.33	142.85	0	0	57.96	61.82	2916.9	850.79	2841.28	0	247.87	325.96	2336.21
35	M	60	MM	not CR (PR)	1	6.47	153.6	6.47	2.68	66.13	359.5	59.02	0	255.69	109.05	64.19	0	464.7	0	0	181.9	0	237.52
36	M	44	MM	not CR (PR)	2	1.79	16.6	3.02	4.69	0	168.07	57.37	0	0	0	0	55.59	560.55	119.63	0	60.27	0	0
37	M	63	MM	not CR (PR)	1	5.8	95.6	5.8	3.4	137.74	139.95	85.37	0	0	53.86	0	84.62	65.6	0	0	0	68.24	117.28
38	M	64	MM	not CR (PR)	2	1.2	18	8.15	8.76	0	0	0	0	0	0	0	455.98	685.24	139.21	0	0	0	0
39	M	36	MM	not CR (PR)	1	8.2	84.2	8.2	6.16	70.1	242.52	59.14	0	0	0	141.16	368.46	1170.16	59.86	0	284.75	344.86	212.35
40	M	54	NHL (MCL)	CR	3	1.14	24.5	3.36	21.89	0	75.04	0	0	63.23	0	0	568.34	1493.02	304.77	0	555.13	494.53	345.13
41	F	60	NHL (MCL)	CR	2	6.7	156.9	10.6	30.34	0	0	0	0	0	0	0	1730.92	1761.87	919.59	0	830.8	816.42	1040.18
42	M	53	NHL (DLBCL)	CR	1	3.9	79.4	3.9	14.34	108.07	313.8	58.73	0	169.29	65.87	65.16	251.05	568.46	60.42	0	182.36	251.52	415.32

Table S1. Clinical characteristics of the patients enrolled in the study along with the SIRT1-SIRT7 expression determined in patients on day 0 and day A.

43	F	62	NHL (DLBCL)	not CR (PR)	2	2.6	46.5	4.42	37.85	262.84	0	77.96	0	124.76	132.1	0	1056.6	2223.17	386.11	0	466.04	1220.01	650.56
44	M	59	NHL (DLBCL)	not CR (PR)	2	1.68	27.18	4.98	16.78	71.25	66.95	0	0	0	61.13	0	202.51	360.01	82.18	0	326.67	176.69	74.21
45	F	42	NHL (HSTL)	not CR (PR)	3	1.85	21.1	3.59	40.5	295.72	425.65	0	0	121.7	106.16	149.3	198.69	1260.5	66.57	0	118.6	237.47	262.37
46	F	60	NHL (ALCL)	not CR (PR)	2	1.65	29.6	5.43	11.25	178.83	132.28	120.69	0	0	0	110.25	124.03	521.84	128.99	0	57.17	51.56	62.81
47	F	38	HL	CR	2	2.8	29.5	4.33	29.8	0	222.69	0	0	66.01	0	0	464.84	1717.12	290.02	0	503.28	546.41	1167.81
48	M	49	HL	not CR (PR)	2	2.26	22.5	5.16	33.16	0	127.77	0	0	136.24	58.04	0	142.24	128.31	129.51	0	199.23	260.79	59.25
49	F	34	HL	not CR (PR)	2	2.3	50.5	5.9	8.08	62.75	0	73.39	0	0	79.59	0	649.44	597.69	439.24	80.66	310.11	785.43	248.6
50	M	21	HL	not CR (PR)	1	12.2	205.5	12.2	42.73	67.22	147.55	0	0	0	116.68	65.45	2215.45	3620.83	1676.18	75.53	1473.39	2404.06	2020.54
