# Supplementary material for: SIRT1-SIRT7 Expression in Patients with Lymphoproliferative Disorders Undergoing Hematopoietic Stem Cell Mobilization
Source: Cancers (Basel). 2022 Feb 25;14(5):1213. doi: 10.3390/cancers14051213 (PMC8909005; doi:10.3390/cancers14051213)
Supplement: Supplementary file 1 [file cancers-14-01213-s001.zip › Table S2.pdf]

Table S2: The PCR thermal cycling conditions (T100 Thermal Cycler, BioRad).

	Cycling step	Temperature, °C	Time	Ramp rate	No of cycles
**Sirt1**	Enzyme activation	95	10 min	2°C/sec	1
	Denaturation	94	30 sec		45
	Annealing/extension	58	1 min		45
	Enzyme deactivation	98	10 min		1
	Hold	4	Infinite		1
**Sirt2**	Enzyme activation	95	10 min	2°C/sec	1
	Denaturation	94	30 sec		45
	Annealing/extension	58	1 min		45
	Enzyme deactivation	98	10 min		1
	Hold	4	Infinite		1
**Sirt3**	Enzyme activation	95	10 min	2°C/sec	1
	Denaturation	94	30 sec		45
	Annealing/extension	58	1 min		45
	Enzyme deactivation	98	10 min		1
	Hold	4	Infinite		1
**Sirt4**	Enzyme activation	95	10 min	2°C/sec	1
	Denaturation	94	30 sec		45
	Annealing/extension	58	1 min		45
	Enzyme deactivation	98	10 min		1
	Hold	4	Infinite		1
**Sirt5**	Enzyme activation	95	10 min	2°C/sec	1
	Denaturation	94	30 sec		45
	Annealing/extension	58	1 min		45
	Enzyme deactivation	98	10 min		1
	Hold	4	Infinite		1
**Sirt6**	Enzyme activation	95	10 min	1°C/sec	1
	Denaturation	94	30 sec		50
	Annealing/extension	58	1 min		50
	Enzyme deactivation	98	10 min		1
	Hold	4	Infinite		1
**Sirt7**	Enzyme activation	95	10 min	2°C/sec	1
	Denaturation	94	30 sec		50
	Annealing/extension	60	1 min		50
	Enzyme deactivation	98	10 min		1
	Hold	4	Infinite		1
