# Supplementary figures and images for: SIRT1-SIRT7 Expression in Patients with Lymphoproliferative Disorders Undergoing Hematopoietic Stem Cell Mobilization
Source: Cancers (Basel). 2022 Feb 25;14(5):1213. doi: 10.3390/cancers14051213 (PMC8909005; doi:10.3390/cancers14051213)

(a)

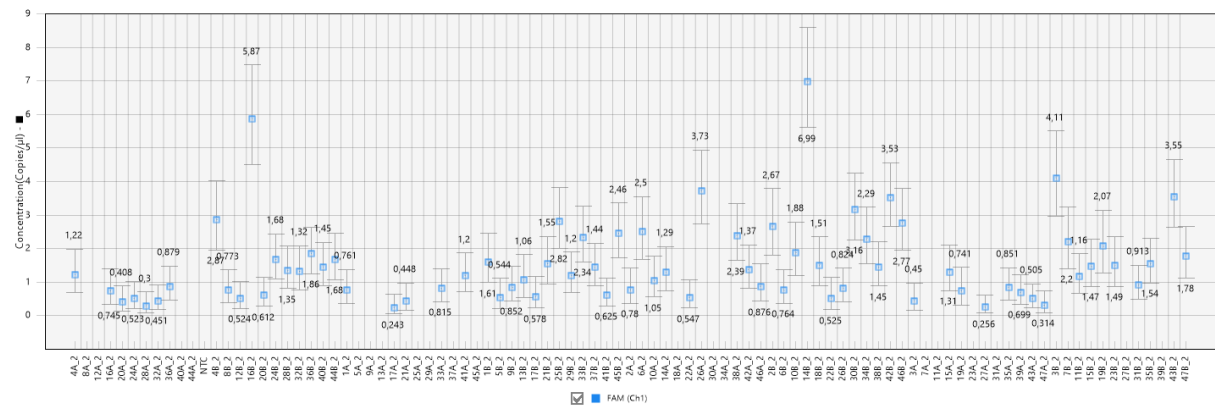

(b)

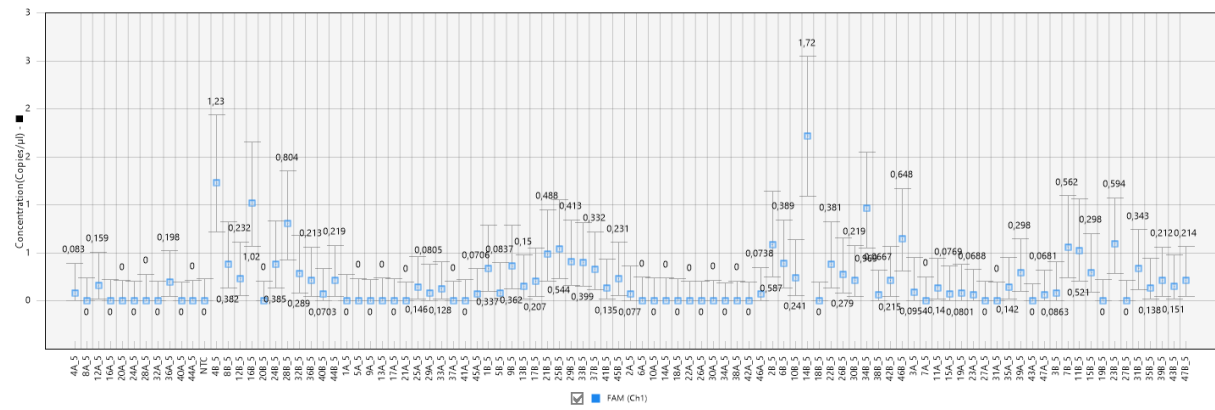

Figure S1: mRNA level [copies/μl] of the (a) SIRT2 and (b) SIRT5.

Supplement: Supplementary file 1 [file cancers-14-01213-s001.zip › Figure S1.pdf]
